# Trends and Patterns in Obesity-Related Deaths in the US (2010–2020): A Comprehensive Analysis Using Centers for Disease Control and Prevention Wide-Ranging Online Data for Epidemiologic Research (CDC WONDER) Data

**DOI:** 10.7759/cureus.68376

**Published:** 2024-09-01

**Authors:** Kosisochi E Achara, Inelefo R Iyayi, Okechukwu C Erinne, Oluwadamilola D Odutola, Uvieroghene P Ogbebor, Stephen N Utulor, Rejoice F Abiodun, Gamamedaliyanage S Perera, Pedro Okoh, Okelue E Okobi

**Affiliations:** 1 Public Health, Emory University, Georgia, USA; 2 Cardiology, WellSpan Cardiology, York, USA; 3 Epidemiology, University of Texas Health Science Center at Houston, Houston, USA; 4 Internal Medicine, Medical University of South Carolina, Florence, USA; 5 Family and Community Medicine, Agape Biomedical Clinic, Abuja, NGA; 6 Family and Community Medicine, Madonna University, Elele, NGA; 7 Medicine, International University Of the Health Sciences (IUHS) School Of Medicine, Basseterre, KNA; 8 Internal Medicine, Spartan Health Sciences University, Vieux Fort, JAM; 9 Family Medicine, Internal Medicine, All Saints University School of Medicine, Roseau, DMA; 10 Emergency Medicine, Lancashire Teaching Hospital, Preston, GBR; 11 Family Medicine, Medficient Health Systems, Laurel, USA; 12 Family Medicine, Lakeside Medical Center, Belle Glade, USA; 13 Family Medicine, Larkin Community Hospital Palm Springs Campus, Miami, USA

**Keywords:** public health intervention, time trends, demographic disparities, icd-10 codes, cdc wonder, obesity mortality

## Abstract

Obesity is a significant public health issue in the United States, contributing to a range of chronic conditions and premature mortality. This study analyzes patterns in obesity-related deaths from 2010 to 2020 using the Centers for Disease Control and Prevention Wide-Ranging Online Data for Epidemiologic Research (CDC WONDER) database to identify trends and demographic disparities. A retrospective analysis was conducted using the CDC WONDER Database, focusing on mortality data associated with specific International Classification of Diseases, Tenth Revision (ICD-10) codes for obesity (E66.0, E66.1, E66.2, E66.8, and E66.9). Data were extracted for the period from January 1, 2010, to December 31, 2020. Mortality rates per 100,000 population were calculated and analyzed across different demographic groups, including age, gender, and race/ethnicity. The analysis revealed an overall increase in obesity-related mortality rates, rising from 1.8 per 100,000 in 2010 to 3.1 per 100,000 in 2020. Age-specific mortality rates showed a significant increase in older age groups, with the highest rates observed in individuals aged 55-64 years (6.4 per 100,000) and 65-74 years (7.2 per 100,000). Gender disparities were evident, with higher mortality rates in males (3.4 per 100,000) compared to females (2.8 per 100,000) by the end of the study period. Racial disparities were also noted, with Black or African American individuals experiencing the highest mortality rates (4.3 per 100,000). The study highlights a concerning upward trend in obesity-related mortality in the United States over the past decade, with notable disparities based on age, gender, and race. These findings underscore the need for targeted public health interventions and policies aimed at reducing obesity prevalence and its associated mortality. Further research should explore the underlying causes and contributing factors to these trends to develop effective strategies for obesity management and prevention. Among the notable strengths of this study include the observation that it leveraged a comprehensive and decade-long countrywide database with detailed and up-to-date ICD-10 codes and demographic data to offer in-depth insights into obesity-related disparities and mortality trends in the United States. Nevertheless, the findings of this study have been limited by its increased focus on the United States' data, depending only on mortality records devoid of consideration of morbidity, alongside the lack of detailed data on lifestyle factors and comorbid conditions.

## Introduction

Obesity or adiposity-based chronic disease is a critical public health issue in the United States, significantly impacting morbidity and mortality rates [1-4]. It is defined as excess body fat that impairs health [1,2]. It is also defined as body mass index (kg/m^2^) ≥ 30, with severe obesity ≥ 40 corresponding to ~100 million United States adults and ~22 million, respectively [2-5]. From 1999 to 2020, the prevalence of obesity increased from 30.5% to 41.9%, even as severe obesity increased from 4.2% to 9.2% [3,5]. This reflects a concerning upward trend over recent decades, highlighting the urgent need to address obesity-related mortality and its public health implications [1-4]. Apart from the impact on an individual's overall health, obesity also presents significant obstacles to healthcare systems and overall productivity in society. Numerous chronic diseases, such as type 2 diabetes, cardiovascular disease, certain malignancies, and respiratory conditions, are more common in obese people [5-8]. In addition to lowering the quality of life, these comorbidities place a heavy financial burden on the healthcare system. For instance, obesity-related medical expenses in the United States are estimated to cost over $170 billion a year [6]. This emphasizes how important it is to have efficient management and prevention plans in place to lessen the effects of obesity [4-6].

The pathophysiology of obesity involves complex interactions between excess adipose tissue and various metabolic processes. The accumulation of excess fat leads to chronic inflammation, insulin resistance, and disruptions in lipid metabolism. Adipose tissue secretes inflammatory cytokines and adipokines, contributing to systemic inflammation and endothelial dysfunction, potentially resulting in cardiovascular disease, hypertension, and type 2 diabetes. Furthermore, obesity increases the risk of conditions such as obstructive sleep apnea and certain cancers. These mechanisms collectively exacerbate the health burden associated with obesity and contribute to elevated mortality rates from obesity-related complications [7-10]. Epidemiological data reveal notable disparities in obesity-related mortality across different demographic groups. Variations in the prevalence of obesity and its complications are observed by age, gender, race, and ethnicity. Research shows that obesity-related mortality rates are higher among older adults and males compared to younger individuals and females [4-7]. In addition, racial and ethnic disparities exist, with Black or African American populations experiencing higher rates of obesity-related deaths compared to other racial groups. Understanding these disparities is essential for designing targeted public health interventions and addressing inequities in healthcare access and outcomes [11]. This study aims to analyze patterns of underlying causes of death due to obesity in the United States among individuals aged 15 years and above, from 2010 to 2020, utilizing data from the Centers for Disease Control and Prevention Wide-Ranging Online Data for Epidemiologic Research (CDC WONDER) database [12]. The objective is to identify trends in obesity-related mortality across various demographic groups and types of obesity, offering insights into evolving patterns over the past decade.

## Materials and methods

Study design

This study employed a retrospective cohort design, analyzing data from 2010 to 2020. Mortality data were extracted for deaths attributed to obesity as an underlying cause. The dataset was stratified by year to capture annual trends and by demographic variables such as age, gender, and race/ethnicity. In addition, data on specific types of obesity, including extreme obesity with alveolar hypoventilation and other obesity, were examined.

Data source

The CDC WONDER database was utilized for this analysis due to its comprehensive collection of mortality data, including detailed information on causes of death, demographics, and medical conditions. The database provides national mortality statistics based on death certificates, which include cause-of-death information coded using the International Classification of Diseases, 10th Revision (ICD-10).

Inclusion and exclusion criteria

The ICD-10 codes E66.0 (obesity due to excess calories), E66.1 (drug-induced obesity), E66.2 (severe obesity with alveolar hypoventilation), E66.8 (other obesity), and E66.9 (obesity, undefined) were the particular categories under which the deaths in this study fell. The data analyzed were limited to the period from January 1, 2010, to December 31, 2020, to capture trends over a decade. Mortality data were disaggregated by age, gender, and race/ethnicity to examine disparities and variations across different population groups. The analysis was restricted to data from the United States that the CDC WONDER database reported, allowing for a focused national trend analysis. Records where obesity was not listed as the underlying cause or where the ICD code did not fall within the specified categories were excluded to maintain a focus on obesity-related mortality. Data with incomplete or missing information, as well as records outside the 2010-2020 time frame, were also excluded to ensure accuracy and relevance.

Data extraction and variables and analysis

Mortality rates per 100,000 population were calculated for each year. The variables of interest included the following: 1) Age groups: Mortality rates were analyzed across different age categories: 15-24 years, 25-34 years, 35-44 years, 45-54 years, 55-64 years, 65-74 years, 75-84 years, and 85+ years. 2) Gender: Separate analyses were conducted for males and females to evaluate gender-based differences in mortality rates. 3) Race/ethnicity: The dataset included racial and ethnic categories: White, Black or African American, Asian or Pacific Islander, and American Indian or Alaska Native. 4) Type of obesity: Two categories were analyzed, namely, extreme obesity with alveolar hypoventilation (categorized as E66.2 under ICD-10 codes) and other obesity (categorized as E66.8 under ICD-10 codes).

Descriptive statistics were used to summarize the data, including calculating annual mortality rates and their 95% confidence intervals (CIs). The rates were derived by dividing the number of deaths attributed to obesity by the total population for each year, then multiplying by 100,000. Confidence intervals for mortality rates were computed to assess the precision of the estimates. Trend analysis was conducted to identify significant changes in mortality rates over time. The analysis included visual inspection of trends and calculation of annual percentage changes. Comparative analysis was performed across different demographic groups and types of obesity to identify disparities and shifts in patterns. All analyses were performed using IBM SPSS Statistics for Windows, version 29.0 (released 2022, IBM Corp., Armonk, NY).

Ethical considerations

The study utilized de-identified data from the CDC WONDER database, ensuring that individual privacy and confidentiality were maintained. As the data were publicly available and anonymized, ethical approval was not required for this analysis.

## Results

From 2010 to 2020, the patterns of underlying causes of death due to obesity in the United States, analyzed using the CDC WONDER database, reveal significant trends across different demographics. The following analysis details the observed mortality rates and trends, including the 95% CI. The overall mortality rate due to obesity increased from 1.8 (1.7-1.8) per 100,000 population in 2010 to 3.1 (3.0-3.2) in 2020. This steady rise over the decade indicates a growing burden of obesity-related deaths. The increase was relatively gradual until 2019, with a sharp spike in 2020, which is attributable to the rising prevalence of cardiovascular disease (including stroke, heart failure, and myocardial infarction) in the age group, a major obesity-associated complication that affects obesity-related health outcomes (Table 1).

**Table 1:** Patterns of underlying causes of death due to obesity (2010-2020)

Mortality rate per 100,000												
Variables	Years	2010	2011	2012	2013	2014	2015	2016	2017	2018	2019	2020
Overall data	Total number of death	5542	5962	6190	6452	6890	7430	7727	7740	7916	8379	10209
	Total population	309M	312M	314M	316M	319M	321M	323M	326M	327M	328M	329M
	Overall mortality rate	1.8 (1.7–1.8)	1.9 (1.9–2.0)	2.0 (1.9–2.0)	2.0 (2.0–2.1)	2.2 (2.1–2.2)	2.3 (2.3–2.4)	2.4 (2.3–2.4)	2.4 (2.3–2.4)	2.4 (2.4–2.5)	2.6 (2.5–2.6)	3.1 (3.0–3.2)
Gender	Male	1.9 (1.8–1.9)	1.9 (1.8–2.0)	2.0 (1.9–2.1)	2.1 (2.0–2.2)	2.2 (2.2–2.3)	2.4 (2.3–2.5)	2.5 (2.4–2.6)	2.5 (2.4–2.6)	2.6 (2.5–2.7)	2.7 (2.6–2.8)	3.4 (3.3–3.5)
	Female	1.7 (1.7–1.8)	1.9 (1.8–2.0)	1.9 (1.9–2.0)	2.0 (1.9–2.0)	2.1 (2.0–2.2)	2.2 (2.2–2.3)	2.3 (2.2–2.4)	2.2 (2.2–2.3)	2.3 (2.2–2.3)	2.4 (2.3–2.5)	2.8 (2.7–2.9)
Race	White	1.8 (1.8–1.9)	1.9 (1.9–2.0)	2.0 (2.0–2.1)	2.1 (2.0–2.1)	2.2 (2.1–2.2)	2.3 (2.3–2.4)	2.4 (2.4–2.5)	2.4 (2.4–2.5)	2.5 (2.4–2.5)	2.6 (2.5–2.7)	3.1 (3.0–3.2)
	Black or African American	2.3 (2.2–2.5)	2.6 (2.5–2.8)	2.5 (2.4–2.7)	2.6 (2.4–2.7)	3.0 (2.8–3.1)	3.1 (3.0–3.3)	3.2 (3.1–3.4)	3.1 (2.9–3.2)	3.1 (3.0–3.3)	3.4 (3.2–3.6)	4.3 (4.1–4.5)
	Asian or Pacific Islander	0.3 (0.2–0.3)	0.2 (0.2–0.3)	0.2 (0.2–0.3)	0.3 (0.2–0.4)	0.2 (0.2–0.3)	0.4 (0.3–0.5)	0.3 (0.3–0.4)	0.3 (0.2–0.4)	0.3 (0.2–0.4)	0.3 (0.3–0.4)	0.6 (0.5–0.7)
	American Indian or Alaska Native	1.8 (1.4–2.3)	1.4 (1.1–1.9)	1.6 (1.2–2.0)	1.6 (1.2–2.0)	1.9 (1.5–2.4)	1.7 (1.4–2.2)	1.9 (1.5–2.3)	1.7 (1.4–2.2)	2.1 (1.7–2.5)	1.6 (1.3–2.0)	2.2 (1.8–2.6)
Age data	15-24 years	0.2 (0.1–0.2)	0.2 (0.1–0.2)	0.2 (0.1–0.2)	0.2 (0.1–0.2)	0.2 (0.2–0.2)	0.2 (0.1–0.2)	0.2 (0.1–0.2)	0.2 (0.1–0.2)	0.2 (0.1–0.2)	0.2 (0.2–0.2)	0.2 (0.2–0.3)
	25-34 years	0.8 (0.7–0.9)	0.9 (0.8–0.9)	0.8 (0.7–0.9)	0.8 (0.7–0.9)	0.9 (0.8–1.0)	0.9 (0.8–1.0)	0.9 (0.8–1.0)	1.0 (0.9–1.1)	0.8 (0.7–0.9)	0.9 (0.8–1.0)	1.2 (1.1–1.3)
	35-44 years	1.8 (1.6–1.9)	1.8 (1.7–2.0)	1.8 (1.6–1.9)	1.8 (1.7–2.0)	2.2 (2.1–2.4)	2.2 (2.0–2.3)	2.3 (2.1–2.4)	2.2 (2.1–2.4)	2.2 (2.1–2.4)	2.2 (2.0–2.3)	2.9 (2.7–3.0)
	45-54 years	2.8 (2.6–3.0)	3.1 (2.9–3.2)	3.1 (3.0–3.3)	3.3 (3.1–3.5)	3.2 (3.1–3.4)	3.7 (3.5–3.9)	3.8 (3.6–3.9)	3.5 (3.3–3.7)	3.6 (3.4–3.7)	3.8 (3.6–4.0)	4.8 (4.6–5.1)
	55-64 years	4.2 (4.0–4.4)	4.2 (4.0–4.5)	4.5 (4.3–4.7)	4.4 (4.2–4.6)	4.6 (4.4–4.8)	4.8 (4.6–5.0)	5.1 (4.8–5.3)	5.1 (4.8–5.3)	5.0 (4.8–5.2)	5.4 (5.2–5.7)	6.4 (6.1–6.6)
	65-74 years	4.5 (4.2–4.8)	4.8 (4.5–5.0)	5.1 (4.8–5.3)	5.3 (5.0–5.6)	5.4 (5.1–5.7)	5.7 (5.4–6.0)	5.7 (5.4–5.9)	5.8 (5.5–6.1)	6.0 (5.7–6.2)	6.0 (5.7–6.3)	7.2 (6.9–7.4)
	75-84 years	3.8 (3.4–4.1)	4.3 (3.9–4.6)	4.2 (3.8–4.5)	4.6 (4.2–4.9)	5.0 (4.6–5.3)	5.3 (4.9–5.7)	5.5 (5.1–5.9)	5.2 (4.8–5.5)	5.7 (5.4–6.1)	6.0 (5.6–6.3)	6.5 (6.1–6.9)
	85+ years	2.4 (2.0–2.8)	2.7 (2.2–3.1)	3.0 (2.5–3.4)	2.7 (2.3–3.1)	2.8 (2.3–3.2)	3.5 (3.0–3.9)	3.5 (3.0–3.9)	3.6 (3.2–4.1)	3.5 (3.0–3.9)	3.9 (3.4–4.4)	4.1 (3.6–4.6)
Types of obesity	Extreme obesity with alveolar hypoventilation	0.04 (0.0–0.1)	0.04 (0.0–0.1)	0.04 (0.0–0.1)	0.04 (0.0–0.1)	0.04 (0.0–0.1)	0.10 (0.0–0.1)	0.05 (0.0–0.1)	0.30 (0.3–0.3)	0.30 (0.2–0.3)	0.30 (0.2–0.3)	0.30 (0.2–0.3)
	Other obesity	1.1 (1.0–1.1)	1.2 (1.1–1.2)	1.2 (1.1–1.2)	1.2 (1.2–1.2)	1.3 (1.2–1.3)	1.4 (1.3–1.4)	1.4 (1.4–1.5)	1.3 (1.3–1.4)	1.4 (1.4–1.4)	1.4 (1.3–1.4)	1.7 (1.7–1.8)

Gender-based trends

**Figure 1:**
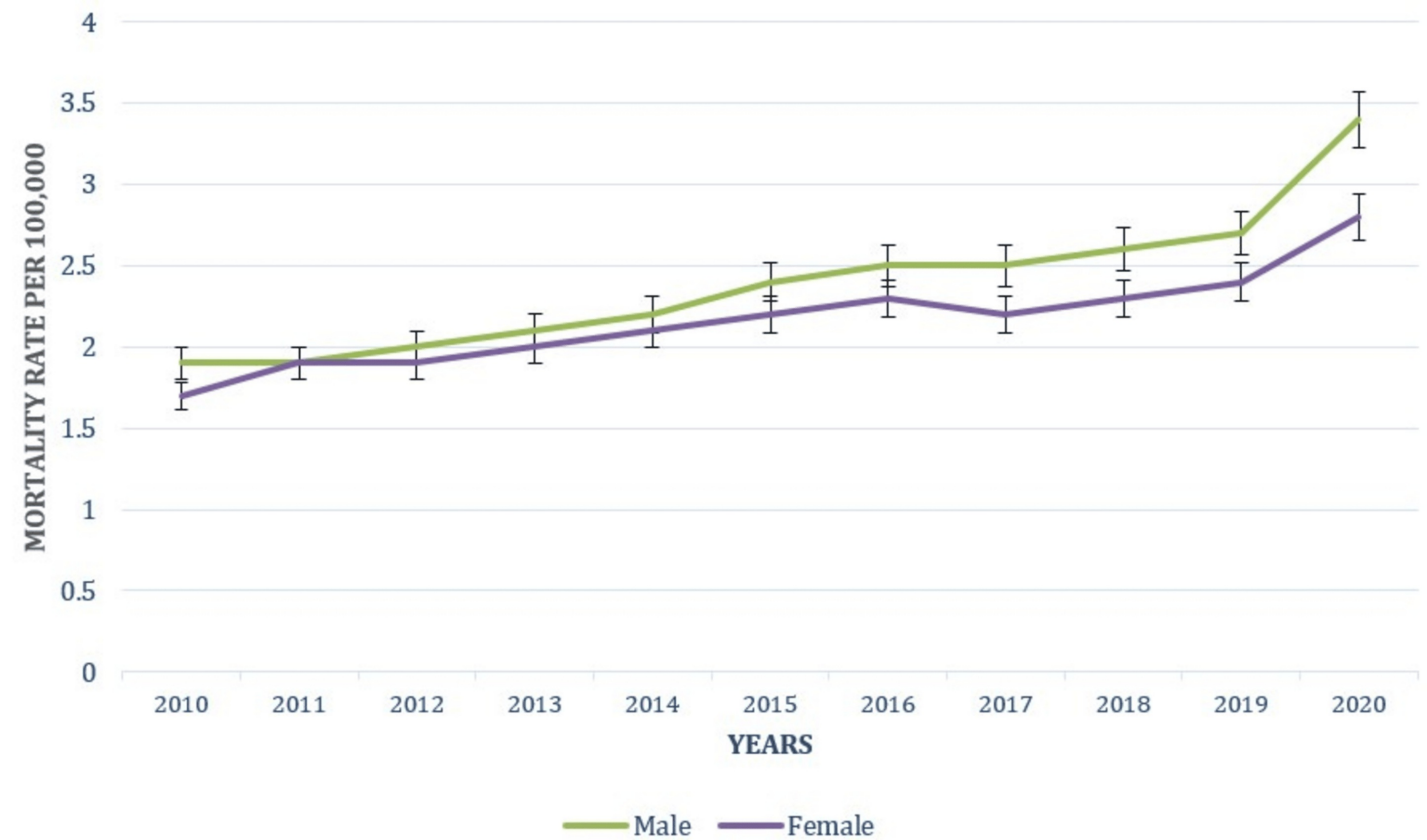
Patterns of underlying causes of death due to obesity based on gender.

The mortality rate for males increased from 1.9 (1.8-1.9) in 2010 to 3.4 (3.3-3.5) in 2020. Males consistently exhibit higher mortality rates compared to females, with a marked rise in the latter years of the study period. This trend underscores the higher risk of obesity-related complications among men. Female mortality rates due to obesity rose from 1.7 (1.7-1.8) in 2010 to 2.8 (2.7-2.9) in 2020. Although females have lower rates compared to males, the increase over the decade is significant and reflects growing obesity-related mortality within this demographic as well. The patterns underlying causes of death attributable to obesity in relation to gender are shown in Figure 1.

Racial and ethnic disparities

**Figure 2:**
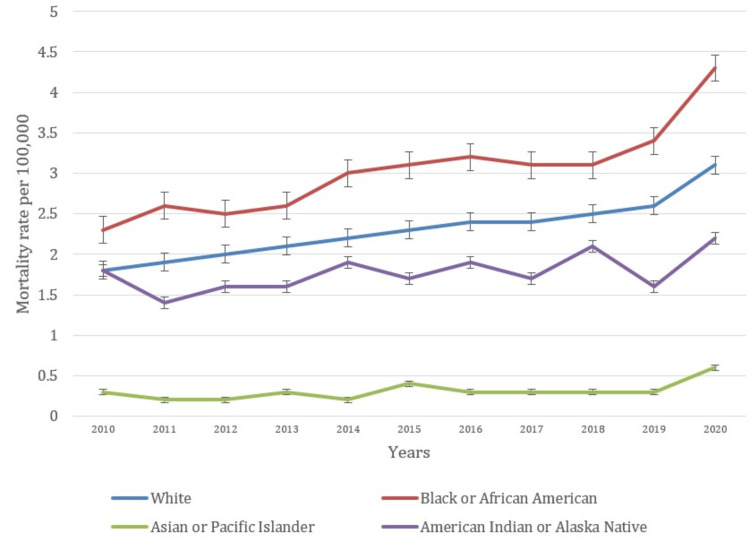
Patterns of underlying causes of death due to obesity based on race

The analysis of obesity-related mortality rates from 2010 to 2020 demonstrates an overall upward trend, with significant increases observed in the final years of the period (Figure 2). This group experienced a steady increase in mortality rates similar to the overall trend, highlighting the growing impact of obesity-related deaths among White Americans. Black or African American groups saw a substantial rise in mortality rates, from 2.3 (2.2-2.5) in 2010 to 4.3 (4.1-4.5) in 2020. The rate of increase is more pronounced compared to other racial groups, indicating a higher relative burden of obesity-related deaths among Black or African American individuals. This disparity points to potential socioeconomic and healthcare access factors affecting this population. Mortality rates for the Asian or Pacific Islander group remained relatively low throughout the period, starting at 0.3 (0.2-0.3) in 2010 and rising to 0.6 (0.5-0.7) in 2020. Although the rates are significantly lower than other racial groups, there is a noticeable upward trend. American Indian or Alaska Native group experienced fluctuations in mortality rates, beginning at 1.8 (1.4-2.3) in 2010 and ending at 2.2 (1.8-2.6) in 2020. The rate variability reflects increases and decreases over the decade, with a recent upward trend. The data also show considerable variation among racial and ethnic groups, emphasizing the need for targeted public health strategies to address obesity and its associated mortality more effectively across different populations. The patterns underlying causes of death attributable to obesity in relation to race are shown in Figure 2.

Age-based trends

**Figure 3:**
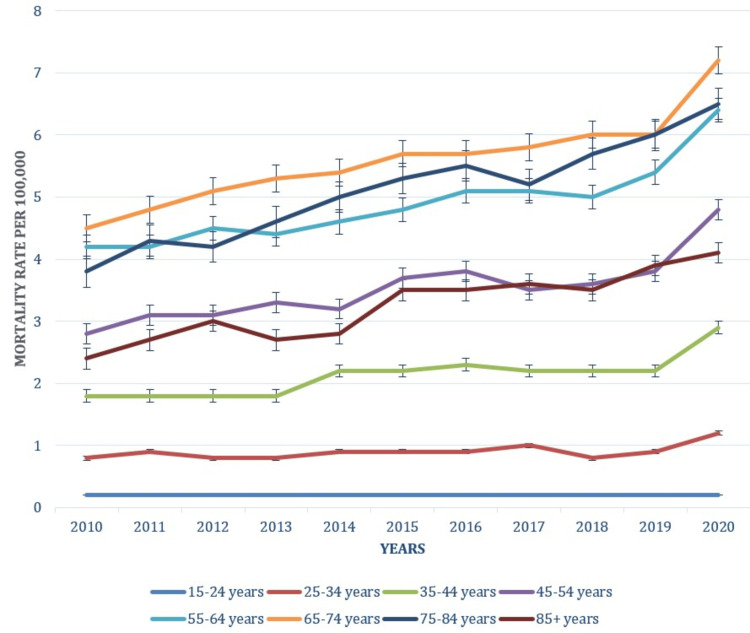
Patterns of underlying causes of death due to obesity based on age

Trends based on age categories are presented in Figure 3. Mortality rates per 100,000 in the 15-24-year age group remained low and stable throughout the period, ranging from 0.2 (0.1-0.2) in 2010 to 0.2 (0.2-0.3) in 2020. The consistency in low rates suggests that obesity-related deaths in younger adults are relatively rare, although the trend shows a slight upward movement toward the end of the period. The 25-34-year group saw an increase in mortality rates per 100,000 from 0.8 (0.7-0.9) in 2010 to 1.2 (1.1-1.3) in 2020. The rate showed a steady rise, peaking in 2020, indicating a growing impact of obesity-related deaths among young adults. In 35-44 years, the age group mortality rates per 100,000 increased from 1.8 (1.6-1.9) in 2010 to 2.9 (2.7-3.0) in 2020. This age group experienced a significant rise in obesity-related deaths over the decade, with a notable spike in recent years, reflecting a higher risk of obesity-related complications in middle-aged adults.

The 45-54-year age group rates per 100,000 rose from 2.8 (2.6-3.0) in 2010 to 4.8 (4.6-5.1) in 2020. This group saw a substantial increase in mortality rates, indicating that middle-aged individuals are increasingly affected by obesity-related deaths. The trend highlights the growing severity of obesity-related health issues as individuals age. The 55-64-year age group experienced an increase from 4.2 (4.0-4.4) in 2010 to 6.4 (6.1-6.6) in 2020. The upward trend is marked, with a consistent rise in mortality rates, reflecting the heightened impact of obesity on older adults. The 65-74-year group mortality rates per 100,000 increased from 4.5 (4.2-4.8) in 2010 to 7.2 (6.9-7.4) in 2020. This age group also saw a significant increase, suggesting that elderly individuals face a higher risk of obesity-related deaths, with the trend accelerating in recent years. The 85+-year age group increased from 2.4 (2.0-2.8) in 2010 to 4.1 (3.6-4.6) in 2020. Despite starting lower, this group experienced a notable rise in mortality rates, underscoring the growing vulnerability of the oldest adults to obesity-related deaths. The patterns underlying causes of death attributable to obesity in relation to age are shown in Figure 3.

Obesity type-based trends

**Figure 4:**
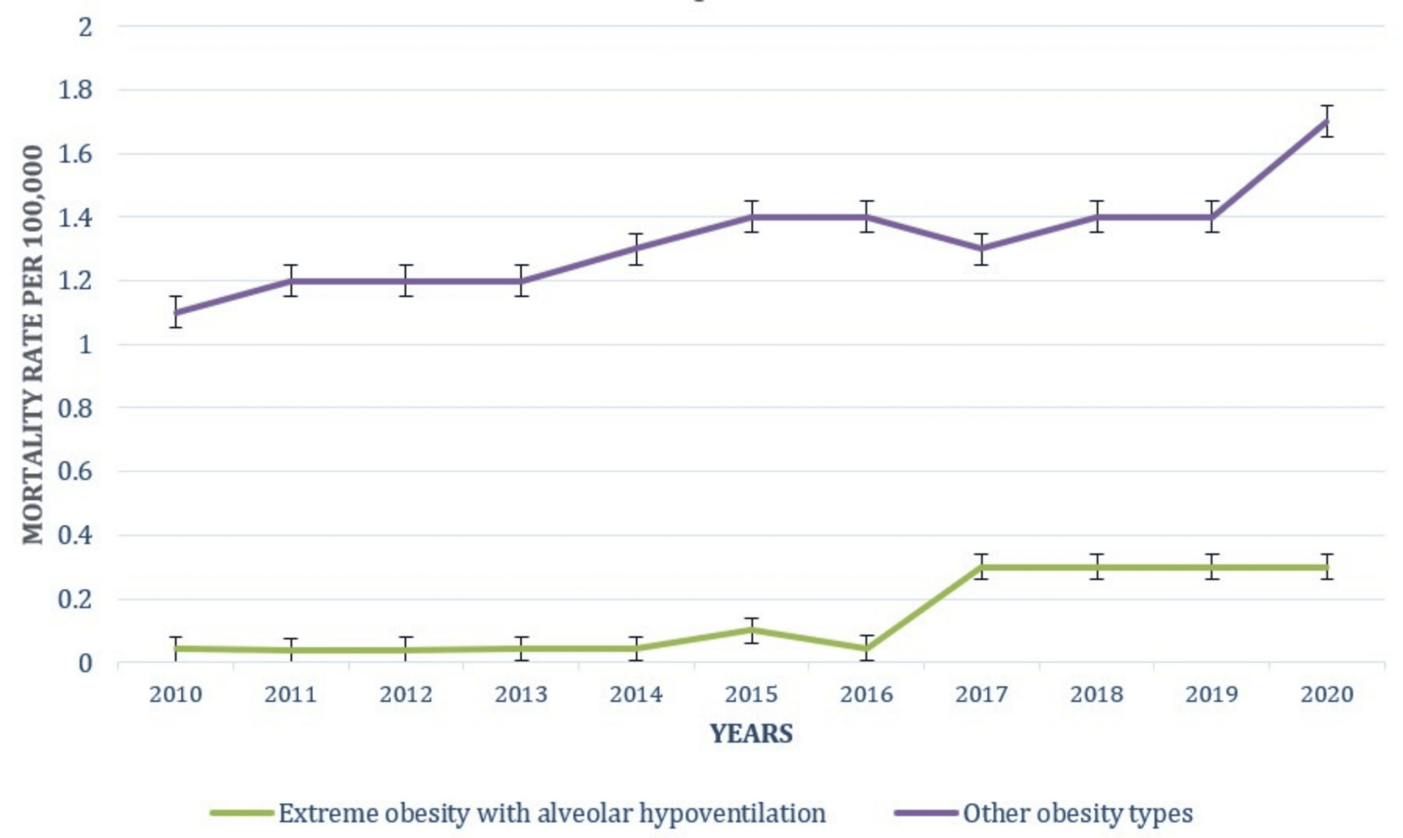
Patterns of underlying causes of death due to obesity based on the obesity type

Trends based on the obesity type are presented in Figure 4. Extreme obesity with alveolar hypoventilation mortality rates of obesity remained relatively low, fluctuating between 0.04 (0.0-0.1) in 2010 and 0.30 (0.2-0.3) in 2020. The rates show a slight increase, with a noticeable spike in 2017, suggesting a growing recognition and impact of this severe obesity complication. The obesity mortality rate for other types of obesity increased from 1.1 (1.0-1.1) in 2010 to 1.7 (1.7-1.8) in 2020. This category showed a consistent rise, reflecting a broader trend of increasing obesity-related deaths not specifically attributed to extreme cases. The patterns underlying causes of death attributable to obesity in relation to obesity are shown in Figure 4.

The data highlight higher mortality rates among older age groups and a pronounced rise in obesity-related deaths. In addition, trends indicate that while younger individuals and those with extreme obesity face relatively lower mortality rates, the overall impact of obesity on mortality is increasing across all demographics. The growing burden underscores the need for targeted public health strategies to address obesity and its associated mortality more effectively.

## Discussion

The patterns of underlying causes of death due to obesity reveal significant trends and disparities across various demographic groups. This discussion interprets these findings, compares them with previous studies, and explores potential implications for public health strategies. Our analysis shows a marked increase in the overall mortality rate due to obesity over the study period. This rise is consistent with the growing recognition of obesity as a major public health issue. Previous research has similarly documented an upward trend in obesity-related mortality. For example, Hales et al. reported a steady increase in both obesity prevalence and associated health risks over recent decades [13]. The sharp spike in mortality rates observed in 2020 aligns with other studies indicating a higher prevalence of cardiovascular disease, which presents that a major complication impacts obesity care health outcomes [13-14].

Our findings highlight that males consistently exhibit higher obesity-related mortality rates compared to females. This trend aligns with previous research suggesting that men face a higher risk of obesity-related complications and mortality. Studies have indicated that obesity-related mortality is generally higher in men, potentially due to differences in fat distribution and associated health risks [15-17]. The observed increase in mortality rates among males underscores the growing severity of obesity-related health issues in this demographic. By contrast, the mortality rate for females due to obesity increased from 1.7 per 100,000 in 2010 to 2.8 per 100,000 in 2020. Although females experience lower rates compared to males, this substantial increase reflects rising obesity trends among women. Previous research has highlighted increasing obesity prevalence and associated health risks among females, necessitating targeted interventions to address obesity in this demographic [15-18].

Significant racial and ethnic disparities in obesity-related mortality were also evident in our study. The mortality rate for Black or African American individuals increased notably from 2.3 per 100,000 in 2010 to 4.3 per 100,000 in 2020. This higher relative burden of obesity-related deaths compared to other racial groups aligns with previous findings by Lofton et al. and Okobi et al., which attributed higher obesity-related mortality rates among Black or African American populations to socioeconomic factors, limited access to healthcare, and higher obesity prevalence [19-20]. By contrast, the mortality rate for White individuals showed a steady rise, similar to the overall trend. This aligns with research by Flegal et al., which observed increasing obesity-related mortality among White populations. Although these rates are lower compared to those of Black or African American individuals, the increasing trend underscores the growing impact of obesity-related deaths across racial groups [21]. Asian or Pacific Islander individuals exhibited relatively low mortality rates, consistent with previous studies suggesting lower obesity-related mortality in this population. Nevertheless, it is noteworthy that, in the Asian/Pacific Islander population, obesity rates might be underestimated based on WHO expert consultation [18]. However, the upward trend observed in this study indicates a need for continued monitoring and intervention [19-21]. The American Indian or Alaska Native population experienced fluctuating mortality rates. Previous research has reported variable trends in obesity-related mortality among this group, reflecting both increases and decreases in obesity prevalence and associated health outcomes. The recent upward trend highlights the need for targeted public health strategies to address obesity-related mortality in this population [13,17-20].

Age-specific mortality rates revealed significant patterns across different age groups. The highest rates were observed in individuals aged 55-64 years, with an increase from 4.2 per 100,000 in 2010 to 6.4 per 100,000 in 2020. This trend aligns with previous research indicating that older adults experience higher obesity-related mortality due to age-related health complications [21]. The upward trend in this age group underscores the necessity for targeted interventions to address obesity and associated health risks among older adults. Conversely, younger age groups, such as those aged 15-24 and 25-34, exhibited lower mortality rates. This trend is consistent with findings by Zheng et al., which reported increasing obesity-related health risks among younger populations [22]. Analysis of specific types of obesity revealed that extreme obesity with alveolar hypoventilation and other forms of obesity both showed increased mortality rates. Extreme obesity with alveolar hypoventilation, although relatively low, demonstrated a noticeable rise, consistent with findings by Antoine et al. indicating increasing mortality related to severe obesity complications [23]. This highlights the need for continued focus on severe obesity and its associated health risks in public health strategies.

Similar studies conducted in recent times have indicated comparable outcomes to our study. For instance, in their study, Min et al. disclosed that obesity-related cardiovascular mortality was increasing in the United States, with differential trends being observed in sex, race, and residential areas [24]. Thus, the study of the population‐level United States mortality data disclosed that the increment in obesity-related cardiovascular and age-adjusted mortality rates was threefold from 2007 to 2020 [24]. Still, the study disclosed that the most widespread major cardiovascular mortality cause associated with obesity included hypertensive diseases and ischemic heart disease and that African Americans presented higher obesity‐linked cardiovascular age‐adjusted mortality rates compared to other races all through the study period [24]. Still, in their study that focused on the impacts on cardiovascular mortality using the Multiple Cause of Death database, Raisi‐Estabragh et al. looked at the increasing prevalence rate of obesity-related cardiovascular mortality, disclosing confounding numbers and clear racial disparities [25]. In addition, similar to our study, this study has demonstrated a significant threefold increment in age-adjusted mortality rates (AAMRs) with regard to obesity-linked cardiovascular mortality between 1999 and 2020, increasing from 2.2 to 6.6 for every 100,000 individuals [25]. Thus, the researchers recorded the highest AAMRs among African Americans, even as Alaska Natives and American Indians were reported to have the most significant increase in AAMRs at 415% [25]. Comparatively, Asian or Pacific Islander, White, and Black people had AAMR increases of 300%, 195%, and 176%, respectively [25].

In addition, similar to the present study, de Cosio et al. evaluated the effects of obesity as a primary cause of mortality in association with comorbid non-communicable diseases, as the contributing causes of death in Black, Hispanic, and White populations in the United States [18]. The study disclosed that the mortality mean was 57.3 years for obesity-related deaths in all groups/races and that individuals dying from obesity were, on average, 15.4 years younger, in comparison to those who died without obesity [18]. Still, similar to our findings, the study disclosed that despite the Hispanic people having higher cardiovascular disease and diabetes prevalence than other groups, they presented the lowest obesity-related mortality rates as the primary cause of death upon association with non-communicable diseases [18]. Furthermore, in evaluating the age at death from different primary causes based on age group and ethnicity/race, the study disclosed that over half of Hispanic and Black people died from various causes at younger ages before attaining 70 years of age, even as nearly 70% of deaths in White people took place after attainment of 70 years [18]. In instances where obesity was the primary cause of mortality, over 85% of Hispanic and Black persons died before attaining 70 years of age in comparison to 78% of White persons. Lastly, the highest rate of obesity-related mortality and related conditions (per one million individuals) for the period between 1999 and 2017 were attributed to cardiovascular disease, cancer, diabetes, and chronic respiratory disease, with Black people presenting the highest mortality rates for all the four non-communicable diseases except cancer and Hispanic people recording the lowest mortality rates for all the four non-communicable diseases [18].

Strength and limitations

This study's strengths include its use of a comprehensive, nationwide database that provides robust and reliable data on mortality. By analyzing a decade-long dataset from 2010 to 2020, the study captures significant trends and variations in obesity-related mortality over time. The inclusion of detailed demographic information, such as age, gender, and race/ethnicity, allows for a nuanced examination of disparities and provides insights into how different population groups are affected by obesity-related deaths. In addition, the focus on specific ICD-10 codes ensures that the analysis is directly related to obesity, enhancing the accuracy of the findings regarding obesity-related mortality. Despite its strengths, this study has several limitations. The study's geographical focus on the United States limits the generalizability of the findings to other countries or regions. In addition, the use of mortality data alone does not account for morbidity associated with obesity, potentially overlooking significant health impacts. The analysis is also limited by the lack of detailed information on comorbid conditions and lifestyle factors, which could provide a more comprehensive understanding of the factors contributing to obesity-related mortality.

Recommendation and future directions

To address the growing burden of obesity-related mortality, public health initiatives should focus on comprehensive strategies to prevent and manage obesity. Implementing targeted interventions for high-risk populations, such as males, Black or African American individuals, and older adults, is crucial. Enhancing public awareness about the health risks associated with obesity and promoting healthy lifestyle choices through educational campaigns can help reduce obesity prevalence. Improving access to healthcare services and resources for obesity management, including screening and treatment options, is essential. Policymakers should consider developing and supporting programs that address socioeconomic disparities and promote equitable access to preventive care and obesity management resources. Future research should explore the underlying causes and contributing factors to the rising obesity-related mortality rates observed in this study. Investigating the impact of socioeconomic status, access to healthcare, and lifestyle factors on obesity-related deaths could provide valuable insights into mitigating strategies. Longitudinal studies examining individual-level data, including comorbid conditions and detailed health histories, would offer a more in-depth understanding of the relationship between obesity and mortality.

## Conclusions

The analysis of obesity-related mortality trends from 2010 to 2020 reveals a concerning upward trajectory, with significant increases observed across various demographic groups. The findings align with previous research, highlighting the growing burden of obesity and its associated mortality. Addressing these trends requires targeted public health interventions focusing on high-risk populations, including racial and ethnic minorities, middle-aged and older adults, and individuals with severe forms of obesity. Future research should continue to monitor these trends and explore effective strategies to mitigate the impact of obesity on public health.
